# Time-Resolved Transposon Insertion Sequencing Reveals Genome-Wide Fitness Dynamics during Infection

**DOI:** 10.1128/mBio.01581-17

**Published:** 2017-10-03

**Authors:** Guanhua Yang, Gabriel Billings, Troy P. Hubbard, Joseph S. Park, Ka Yin Leung, Qin Liu, Brigid M. Davis, Yuanxing Zhang, Qiyao Wang, Matthew K. Waldor

**Affiliations:** a State Key Laboratory of Bioreactor Engineering, East China University of Science and Technology, Shanghai, China; b Qingdao National Laboratory for Marine Science and Technology, Qingdao, China; c Department of Microbiology and Immunobiology and Brigham and Women’s Hospital, Harvard Medical School, Boston, Massachusetts, USA; d Howard Hughes Medical Institute, Boston, Massachusetts, USA; e Department of Biology, Trinity Western University, British Columbia, Canada; Harvard School of Public Health

**Keywords:** *Edwardsiella piscicida*, live attenuated vaccine, pattern analysis of conditional essentiality (PACE), transposon insertion sequencing, fitness dynamics and profiles

## Abstract

Transposon insertion sequencing (TIS) is a powerful high-throughput genetic technique that is transforming functional genomics in prokaryotes, because it enables genome-wide mapping of the determinants of fitness. However, current approaches for analyzing TIS data assume that selective pressures are constant over time and thus do not yield information regarding changes in the genetic requirements for growth in dynamic environments (e.g., during infection). Here, we describe structured analysis of TIS data collected as a time series, termed pattern analysis of conditional essentiality (PACE). From a temporal series of TIS data, PACE derives a quantitative assessment of each mutant’s fitness over the course of an experiment and identifies mutants with related fitness profiles. In so doing, PACE circumvents major limitations of existing methodologies, specifically the need for artificial effect size thresholds and enumeration of bacterial population expansion. We used PACE to analyze TIS samples of *Edwardsiella piscicida* (a fish pathogen) collected over a 2-week infection period from a natural host (the flatfish turbot). PACE uncovered more genes that affect *E. piscicida*’s fitness *in vivo* than were detected using a cutoff at a terminal sampling point, and it identified subpopulations of mutants with distinct fitness profiles, one of which informed the design of new live vaccine candidates. Overall, PACE enables efficient mining of time series TIS data and enhances the power and sensitivity of TIS-based analyses.

## IMPORTANCE

Transposon insertion sequencing (TIS) enables genome-wide mapping of the genetic determinants of fitness, typically based on observations at a single sampling point. Here, we move beyond analysis of endpoint TIS data to create a framework for analysis of time series TIS data, termed pattern analysis of conditional essentiality (PACE). We applied PACE to identify genes that contribute to colonization of a natural host by the fish pathogen *Edwardsiella piscicida.* PACE uncovered more genes that affect *E. piscicida*’s fitness *in vivo* than were detected using a terminal sampling point, and its clustering of mutants with related fitness profiles informed design of new live vaccine candidates. PACE yields insights into patterns of fitness dynamics and circumvents major limitations of existing methodologies. Finally, the PACE method should be applicable to additional “omic” time series data, including screens based on clustered regularly interspaced short palindromic repeats with Cas9 (CRISPR/Cas9).

## INTRODUCTION

The coupling of transposon mutagenesis with high-throughput sequencing of transposon insertion sites enables comprehensive mapping of the genetic determinants of bacterial fitness (i.e., the extent to which individual loci contribute to survival and/or growth). In high-density transposon insertion libraries, insertion frequency at each locus is generally inversely correlated with the locus’s contribution to *in vitro* fitness. Furthermore, a locus’s contribution to fitness under a selective condition can be inferred from changes in the relative abundance of corresponding mutants following the imposition of a selective pressure (e.g., passage of a transposon library through an animal model of infection). These two principles underlie a variety of methodologically related approaches to transposon insertion sequencing (TIS [e.g., TnSeq, INSeq, TraDIS, or HITS]), which have been used to define genes required for viability and for optimal fitness under one or more selective conditions (essential and conditionally essential [CE] loci, respectively) (1–7).

Virtually all TIS studies have assessed genetic contributions to fitness in a particular environment based on observations at a single sampling point. The most basic approach evaluates relative fitness based on “fold change” (FC), a ratio of a mutant’s abundance in the library before and after selection. However, there are three problematic aspects of this approach. First, such analyses routinely impose an effect size threshold (i.e., a particular FC value) beyond which fitness alterations are considered “significant”; consequently, classification of mutants is dependent upon the somewhat arbitrary selection of this threshold. Second, comparative analyses of effect sizes across different experimental conditions can be challenging, since the FC for each locus is influenced not only by its contribution to fitness but also by the duration of the experimental selection. Finally, reliance on a single endpoint FC to identify and classify loci fails to capture potentially dynamic selective pressures that mutants may encounter in an experimental system (e.g., an animal model of infection).

van Opijnen et al. (4, 5) developed a more rigorous approach in which changes in a mutant’s abundance are considered within the context of expansion of the bacterial population. A mutants’ fitness costs are calculated per generation, which effectively normalizes for the duration of selection. Relative fitness can then be expressed in terms of each mutation’s effect on growth rate. By focusing on growth rate, this approach minimizes complications associated with arbitrary selection of an effect size threshold and facilitates interexperimental comparisons. However, it is reliant upon accurate quantification of bacterial population expansion, which can be challenging in some experimental systems (e.g., animal models of infection), and it rests on the assumption noted above that selective pressures are static throughout the course of the experiment.

*Edwardsiella piscicida* (formerly included in *Edwardsiella tarda* [8–10]) is a facultative, intracellular pathogen and one of the chief infectious threats to farm-raised fish (11). *E. piscicida* is also an opportunistic pathogen of humans (12). The pathogen is resistant to multiple antibiotics (9), limiting treatment options for the aquaculture industry. In a turbot (*Scophthalmus maximus* L.) infection model, *E. piscicida* pathogenicity has been shown to depend on both its type III secretion system (T3SS) and type VI secretion system (T6SS) (12–15). Furthermore, both of these virulence-associated secretion systems require a common factor, EsrB, for their expression (14, 16, 17). However, there is little knowledge about how these virulence-associated secretion systems enable infection or of additional *E. piscicida* virulence factors.

Here, we build upon existing methodologies for analysis of endpoint TIS data by creating a framework (pattern analysis of conditional essentiality [PACE]) for analysis of time series TIS data that enables definition of dynamic bacterial fitness requirements over the course of an experiment. We demonstrate the utility of this approach by analyzing *E. piscicida* colonization of a natural fish host, the flatfish turbot.

## RESULTS

### TIS analysis and validation of genes required for host colonization.

**FIG 1:**
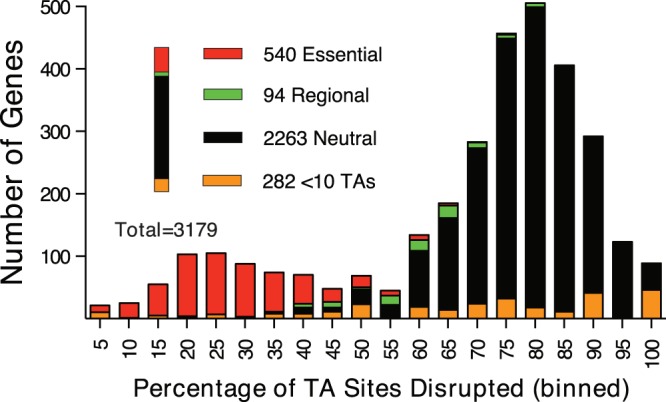
Histogram showing the percentage of TA sites disrupted per gene in the *E. piscicida* transposon insertion library. Genes containing more than 10 TA sites were further classified using the EL-ARTIST analysis pipeline as either essential, regionally essential (regional), or neutral.

To identify protein-coding loci required for growth of *E. piscicida in vitro*—a useful precursor for analyses of *in vivo* growth—we used a mariner-based Himar1 transposon to generate a high-density transposon insertion library in *E. piscicida* EIB202 (8) and then determined the genomic distribution of insertion sites using massively parallel sequencing. The distribution of insertion sites was further characterized using EL-ARTIST, a hidden Markov model (HMM) analysis pipeline that identifies loci statistically underrepresented among insertion mutants (18). Sequencing of the *E. piscicida* library identified 80,616 distinct insertion mutants (57.19% of TA sites, for which Himar1 has sequence specificity). As expected for a highly complex library approaching saturation of insertion sites (19), a histogram of the percentage of TA sites disrupted per gene contained two peaks (Fig. 1). The major peak, centered at 80%, consists largely of genes classified as “neutral” by EL-ARTIST (i.e., lacking an effect on fitness); the center of this distribution reflects the average percentage of sites disrupted within genes dispensable for viability. The minor peak, centered at ~25%, is dominated by genes classified as “essential” for *in vitro* growth due to the dearth of associated insertions (see Table S1 in the supplemental material). The 673 loci classified by EL-ARTIST as either “essential” or “regional” (i.e., lacking insertions within a portion of the coding sequence) are disproportionately associated with biological processes that are also required for the growth of related organisms *in vitro* (see Fig. S1 in the supplemental material) (19, 20). Collectively, these attributes suggest the insertion library—the first reported for *E. piscicida*—is sufficiently complex to enable robust comparative analyses with the *in vivo*-passaged libraries obtained during our subsequent time series analyses.

10.1128/mBio.01581-17.2 FIG S1  COG categories that show statistically significant enrichment and depletion among regional and underrepresented genes in the *E. piscicida in vitro* (input) library. The normalized representation is the observed number of regional and essential genes per category (“obs”) relative to the expected number per category based on random sampling of all genes (“sim”). (The genes with a TA number higher than 10 were taken into account.) Download FIG S1, PDF file, 0.1 MB. Copyright © 2017 Yang et al. 2017 Yang et al. This content is distributed under the terms of the Creative Commons Attribution 4.0 International license.

10.1128/mBio.01581-17.6 TABLE S1  EL-ARTIST analysis of the TIS libraries. Download TABLE S1, XLS file, 0.5 MB. Copyright © 2017 Yang et al. 2017 Yang et al. This content is distributed under the terms of the Creative Commons Attribution 4.0 International license.

We optimized an established model of turbot infection (21) by varying inoculum sizes in order to identify an *E. piscicida* dose likely to result in host survival kinetics compatible with time series analysis as well as maintenance of sufficient library complexity for TIS studies. Intraperitoneal (i.p.) injection of 3 × 10^6^ CFU (~2 × 10^4^ CFU per g of fish body weight) of *E. piscicida* was found to result in limited mortality at the desired endpoint of 14 days postinfection (dpi) (see Fig. S2A in the supplemental material), despite robust colonization of turbot livers and spleens (>10^4^ CFU/g) at 1 dpi and kidneys at 2 dpi, and 100% mortality by 28 dpi (Fig. S2A and B). Consequently, this dose (which corresponds to ~40× library coverage) was administered for subsequent infections using the *E. piscicida* insertion library.

10.1128/mBio.01581-17.3 FIG S2  Survival and bacterial load in turbot injected with various doses of *E. piscicida*. (A) Survival of turbot i.p. inoculated with the indicated doses of WT *E. piscicida* EIB202 or with PBS. Means with SEM are shown (*n =* 3). (B) Bacterial load (CFU/g tissue) in turbot liver, kidney, and spleen at various time points after i.p. injection of the indicated doses of *E. piscicida* EIB202 (*n =* 5 per time point). Download FIG S2, PDF file, 0.1 MB. Copyright © 2017 Yang et al. 2017 Yang et al. This content is distributed under the terms of the Creative Commons Attribution 4.0 International license.

**FIG 2:**
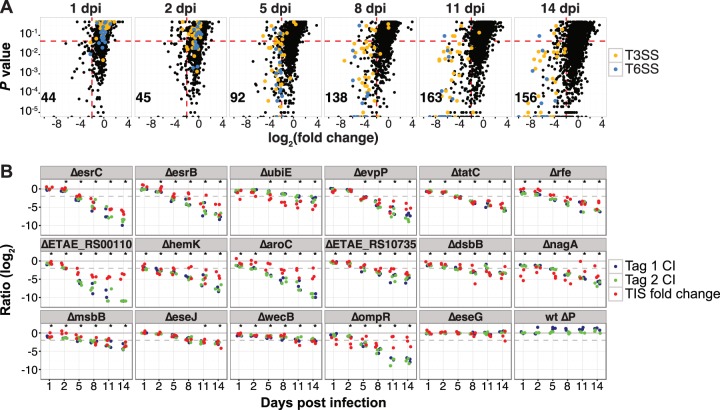
TIS time series results from turbot infection studies and validation of selected *in vivo*-attenuated mutants. Bacteria were recovered from fish livers at the indicated time points and analyzed via high-throughput sequencing of transposon insertion sites or barcode tags. (A) Data from one of three replicate transposon insertion libraries are depicted for each time point. *P* values produced for each locus, using a Mann-Whitney *U* test, were plotted against mean log_2_ fold change (FC [i.e., the log_2_-transformed value of the ratio of normalized output versus input reads]). Red dashed lines represent the thresholds for CE loci (*P* < 0.05; log_2_ FC, <−2). In the lower left corner, the number of genes meeting both criteria in two of three library replicates is indicated. (B) The barcoded WT, wt_ΔP, and in-frame deletion mutant strains were recovered from fish inoculated with a pool of WT and mutant strains, and competitive indices (CIs) were calculated based on the ratios of individual mutant to WT tags in output versus input. The *y* axis shows the log_2_-transformed ratio of either CI (blue and green dots) or TIS (red dots) FC values. The gray dashed line shows *y* = −2. *, *P* ≤ 0.01 for CI results, based on one-way ANOVA followed by Dunnett’s test for multiple comparisons. Mutants are ranked based on FC in TIS data at 14 dpi.

For the TIS analyses, turbot were i.p. infected with 3 × 10^6^ CFU of the *E. piscicida* library, and bacteria were recovered from the livers of infected fish at 1, 2, 5, 8, 11, or 14 dpi. Five livers were pooled for each time point in each of 3 infection cohorts. TIS of the recovered libraries, coupled with comparisons of the normalized read count per locus, revealed that the 3 biological replicate libraries per time point all had pairwise coefficients (*r*^2^; see Materials and Methods) higher than 0.8, suggesting that pooled infections yielded highly reproducible results (see Fig. S3 in the supplemental material) and thus that the experimental design did not appear compromised by infection bottlenecks or other factors that might limit library complexity. Comparative analyses were also performed between normalized read count per locus in output libraries versus the input library. Genes with relative read counts that declined significantly during the experiment were identified using a Mann-Whitney *U* test (MWU [log_2_ FC of <−2; MWU *P* value of <0.05]) (see Table S2 in the supplemental material). By 14 dpi, 156 genes met the selected effect size threshold, which is often imposed to define conditional essentiality (CE) (20). These included almost all genes associated with *E. piscicida*’s type III (29 of 34) and type VI (15 of 16) secretion systems (T3SS and T6SS, respectively) (8), which are known to be important for pathogenesis (Fig. 2A; Table S2) (12–16), as well as a few unrelated loci previously linked to *E. piscicida* virulence (e.g., *rfe* and *tatA* to *-E*) (22). Fewer loci, including a smaller subset of T3SS and T6SS loci, met selective criteria at earlier time points, illustrating how identification of CE loci is dependent on a combination of the effect size threshold and the duration of selection.

10.1128/mBio.01581-17.4 FIG S3  Similarity of output libraries recovered from livers of infected animals. The normalized reads per locus (including all genes and intragenic regions) were plotted for libraries recovered following injection of each of the three input libraries (biological replicates). Comparisons were made for libraries recovered at 1, 2, 5, 8, 11, and 14 dpi. Pairwise correlation coefficients (*r*^2^) are indicated. Download FIG S3, PDF file, 1.5 MB. Copyright © 2017 Yang et al. 2017 Yang et al. This content is distributed under the terms of the Creative Commons Attribution 4.0 International license.

10.1128/mBio.01581-17.7 TABLE S2  Conditional essential genes identified in liver by 14-day endpoint TIS analysis. Download TABLE S2, XLS file, 0.1 MB. Copyright © 2017 Yang et al. 2017 Yang et al. This content is distributed under the terms of the Creative Commons Attribution 4.0 International license.

To confirm that loci had been accurately classified as CE in this infection model, we generated barcoded deletion mutants of 16 putative CE genes (see Table S3A in the supplemental material), including several associated with T3SS or T6SS, and measured their competitive indices (CIs) when coinjected along with a barcoded wild-type (WT) strain and two mutant strains known to be proficient at colonization, the wt_ΔP strain (which lacks plasmid pEIB202) and the Δ*eseG* strain (23, 24), which served as controls. All of the putative CE loci exhibited progressive declines in their respective CIs, whereas the control strains did not (Fig. 2B), confirming the reliability of the TIS analysis. Moreover, there was excellent concordance between the progressive declines in the CIs and the gradual reduction in insertion mutants observed in the TIS data (Fig. 2B, compare green and blue dots [tagged mutants] with red dots [TIS data]). Thus, the validation studies suggested that the time series TIS output comprised a robust and reliable data set that could be used for developing an analytic framework to decipher temporal patterns in fitness dynamics.

10.1128/mBio.01581-17.8 TABLE S3  (A) List of strains used for CI experiments. (B) Bacterial strains and plasmids used in this study. (C) Primers used in this study. Download TABLE S3, XLS file, 0.1 MB. Copyright © 2017 Yang et al. 2017 Yang et al. This content is distributed under the terms of the Creative Commons Attribution 4.0 International license.

### Serial model fitting of time series TIS data identifies genes contributing to *in vivo* fitness.

To describe the fitness of the mutants in our library over time, we developed an analytic framework called PACE (pattern analysis of conditional essentiality). PACE begins by modeling the abundance of each mutant with a polynomial equation, using serial model refinement to identify the optimal model. We then use the polynomial coefficients thus determined to cluster mutants that display similar changes in abundance with time. A similar approach (using linear splines rather than polynomials) has been previously applied to proteomic and transcriptomic data sets (25).

**FIG 3:**
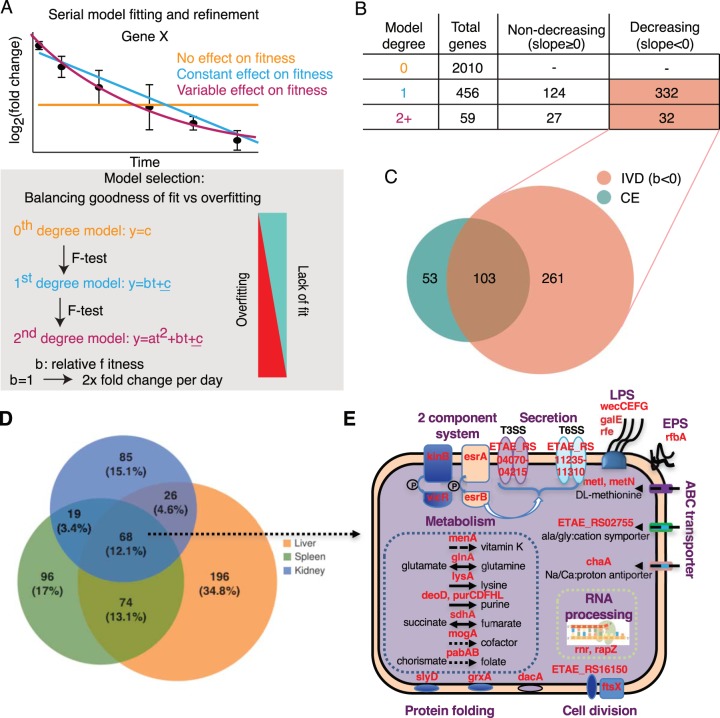
Analysis of time series TIS data via PACE. (A) Schematic representation of curve fitting within PACE. Relative abundance curves for each gene are fit to a series of models of increasing polynomial degree, and models are selected based on an *F* test of nested models, balancing goodness of fit and overfitting. (B) PACE results for *E. piscicida* isolated from livers of infected fish, showing the distribution of genes among the different polynomial models. Note that “nondecreasing” here includes genes with negative slope, but for which a 95% confidence interval of the slope included zero; these are excluded from the *in vivo* decreasing (IVD) group. (C) A Venn diagram compares genes classified as conditionally essential (CE) based on endpoint analysis (*P* < 0.05; log_2_ FC, <−2) and genes assigned to the IVD category based on assignment by PACE of a relative fitness value (*b*) of <0. (D) Venn diagram comparing IVD genes in the liver, spleen, and kidney. (E) Functional classification of the 68 genes classified as IVD in all 3 organs.

For each gene, PACE fits the *in vivo* TIS time series data (log-transformed abundance relative to the input library at each time point) to a series of polynomial models of increasing degree (Fig. 3A). Because we have relatively few time points for each locus, and because of the intrinsic measurement noise, overfitting is a significant concern; consequently, an *F* test is used for each polynomial degree to determine if increasing the complexity of the model is justified (see Materials and Methods). We expect that most loci will exhibit a steady abundance over time and consequently are best fit by a constant (zero-degree) polynomial. Genes fit by zero-degree models are consistent with a uniform log FC over time, indicating that disruption of the gene has no effect upon fitness during the interval assayed. For genes fit by first-degree equations, the log-transformed FC values appear to change with a constant rate (i.e., plots are linear with nonzero slope), reflecting a constant fitness benefit/defect associated with gene disruption. Finally, genes whose disruption has variable effects upon fitness over the course of the experiment are best fit by higher-degree polynomials involving additional coefficients (e.g., Fig. 3A, coefficient *a*).

Application of PACE to the TIS data of *E. piscicida* recovered from the livers of infected turbot revealed that 2,010 loci were best fit by zero-order models (out of the 2,525 classified as “neutral” and “regional essential” *in vitro* [Table S1] and having sufficient representation *in vivo* for analysis, which was defined as being represented in at least 25% of samples and having at least 50 reads in the input library). These 2,010 loci either have little effect on *in vivo* fitness during all time series or have TIS data that are sufficiently noisy to preclude fitting to a more complex model. Of the remaining 515 loci, 456 were best fit by first-degree equations, 58 were best fit by second-degree equations, and 1 corresponded to a third-degree equation (Fig. 3B). We were particularly interested in the 364 nonzero-order genes modeled with a negative linear coefficient (i.e., *b* < 0), for which the 95% confidence interval excluded positive values. These 364 genes, which we termed “*in vivo* decreasing” (IVD), are presumed to be necessary for optimal growth during the experimental time frame (Fig. 3B; see Table S4 in the supplemental material). Thus, we are defining genes as having a fitness defect during the course of the experiment based on the linear coefficient. The set of IVD loci included all 32 genes from the T3SS that passed our initial filter, as well as all 16 from the T6SS. Other known virulence genes in the IVD group included *tatA*, *tatB*, and *tatC* (22), which were also classified as CE (Table S2). Comparison of CE (endpoint-based selection) and IVD (fitness pattern-based selection) genes showed that the IVD group included most of the CE loci, but also encompassed many additional genes that did not meet the effect size criteria for CE analysis (Fig. 3C). The relatively small set of loci classified as CE but not IVD consisted predominantly of genes that either did not pass our initial triage due to lack of data or were significantly attenuated relative to input on day 1 while not changing in abundance over the course of the experiment; the latter genes may be critical to early survival *in vivo* rather than subsequent proliferation, but further study is needed to clarify their roles.

10.1128/mBio.01581-17.9 TABLE S4  IVD genes and their PACE clustering in various tissues. Download TABLE S4, XLS file, 0.2 MB. Copyright © 2017 Yang et al. 2017 Yang et al. This content is distributed under the terms of the Creative Commons Attribution 4.0 International license.

While most studies of *E. piscicida* pathogenicity have focused on the liver, the pathogen also colonizes the spleen and kidney following i.p. inoculation. Thus, we also performed time series TIS analysis of *E. piscicida* samples recovered from the spleens and kidneys of infected animals. Although *E. piscicida* colonization of the kidney was generally lower than that of the spleen and liver, it was sufficient to enable recovery of representative samples of the passaged library from all time points except 1 dpi. In total, 68 genes were classified as IVD in all 3 organs (Fig. 3D; Table S4). These consensus genes are required for the biosynthesis of the *E. piscicida* T3SS and T6SS, lipopolysaccharide (LPS), purines, certain amino acids, cofactors, and vitamin K (Fig. 3E), suggesting that these secretion systems and biosynthesis pathways are common requirements for the pathogen’s fitness in all three organs or general fitness within the host. In contrast, most genes that were identified as IVD in one organ were organ specific and therefore may mediate bacterial processes that are differentially required for fitness in specific host tissues.

### Clustering of time series curves reveals distinct patterns of *in vivo* fitness.

**FIG 4:**
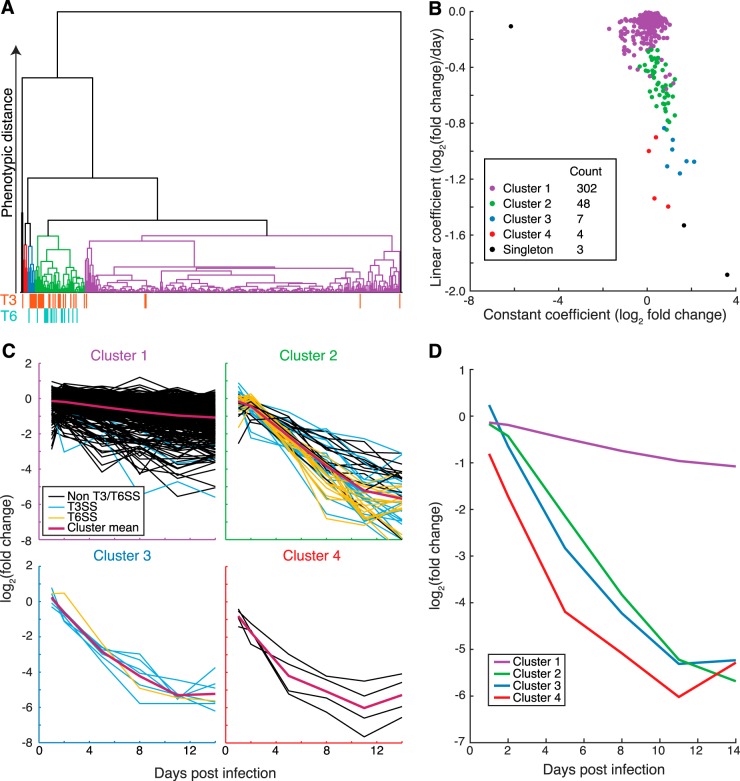
PACE enables identification of gene clusters exhibiting similar *in vivo* dynamics. (A) Dendrogram of IVD loci hierarchically clustered according to their fitting coefficients and colored according to cluster, with all singleton clusters colored black. The clustering cutoff was selected to identify clusters enriched for T3SS/T6SS genes. (B) Distribution of constant (*x* axis) and linear (*y* axis) coefficients of IVD genes, colored according to the clustering in panel A. (C) Relative abundance time series curves for each cluster, showing data for each gene (mean over *n =* 3 biological replicates) along with the cluster mean (magenta). T3SS and T6SS genes are highlighted in blue and yellow, respectively. (D) Relative abundance time series for each non-singleton cluster in panel C; at each time point, the mean of all genes in each cluster is plotted.

To further exploit the output of model fitting, we performed hierarchical clustering of hepatic IVD loci based on each gene’s fitting coefficients (i.e., constant, linear, and quadratic), in order to identify loci that exhibit similar *in vivo* dynamics. We carried out this analysis on data derived from liver samples because this organ had the most robust colonization throughout the experiment. Because we hypothesized that genes with biologically related roles ought to display similar behaviors and be grouped together, we selected clustering cutoffs that maximized the number of clusters while keeping T3SS- and T6SS-linked mutants (known to have similar phenotypes) grouped together (Fig. 4A). Our analysis grouped IVD loci into 4 clusters and 3 singleton loci (Fig. 4A; clusters are color coded) whose distinct attributes are evident when genes are plotted (Fig. 4B) based on their equations’ constant terms and linear coefficients. The majority of genes fell into cluster 1 with a median fitness defect of −0.074 log_2_ FC/day (95% confidence interval, −0.081 to −0.069); this cluster was comprised largely of genes not classified as CE, but which nonetheless clearly exhibited a gradual decline in associated mutants during the course of the infection (Fig. 4C). Clusters 2 and 3 exhibited markedly greater fitness defects: medians were −0.50 log_2_ FC/day (95% confidence interval, −0.57 to −0.42) and −1.07 log_2_ FC/day (95% confidence interval, −1.11 to −0.92), respectively. Clusters 2 and 3 were highly enriched for T3SS and T6SS genes, consistent with the known requirement for such genes during *in vivo* growth (Fig. 4C) (12–16). Cluster 2 also included several additional loci previously linked to virulence, including *tatC*, *wecEF*, and *rfe* (Table S4) (21, 26). Finally, the small cluster, cluster 4 (4 genes), was associated with large fitness defects and comparatively low initial abundance (relative to cluster 3 genes, which have a similar fitness defect [see Tables S4 and S5 in the supplemental material]). Notably, cluster 2, 3, and 4 mean log_2_ FC values converge by day 14 (Fig. 4D); consequently, endpoint-based analyses cannot be used to distinguish between these clusters.

10.1128/mBio.01581-17.10 TABLE S5  Raw sequencing read numbers. Download TABLE S5, XLS file, 1.2 MB. Copyright © 2017 Yang et al. 2017 Yang et al. This content is distributed under the terms of the Creative Commons Attribution 4.0 International license.

### Using fitness patterns to guide development of LAV candidates.

Attenuated *E. piscicida* strains would be a boon for the aquaculture industry as potential live attenuated vaccine (LAV) candidates (11). Notably, among mutants previously developed as *E. piscicida* LAV strains, all have one or more disruptions within genes that were found in the cluster 2 pattern of attenuation (e.g., mutants lacking *aroC* [21], *esrB* [14, 27, 28], *tatC* [22], or the multideletion WED strain, which lacks *aroC* and T3SS components [21]). Thus, we reasoned that the fitness dynamics associated with strains in cluster 2 are well suited for LAV and that our gene clustering results might aid development of novel vaccine strains. Since an *esrB* mutant, which inactivates both T3SS and T6SS (16, 17), has already been tested as an LAV (14), we focused on the 13 genes in cluster 2 that are not components of the T3SS or T6SS clusters as alternative candidates for disruption in testing new *E. piscicida* LAV strains.

**FIG 5:**
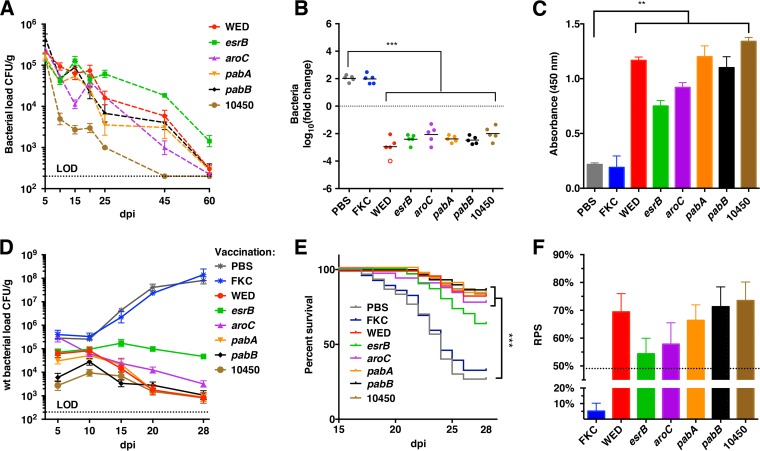
Efficacy of new live attenuated vaccine strains identified based on PACE. (A) Bacterial loads recovered from kidneys of fish inoculated i.p. with the LAV candidates at a dose of 3 × 10^5^ CFU/fish. Each time point reflects the mean and standard error of the mean (SEM) from 5 fish. The dotted line indicates the limit of detection (LOD [200 CFU/g]). (B) Bactericidal capacities of sera from turbot inoculated with the indicated LAV candidates or controls. WT *E. piscicida* cells were incubated at 30°C for 8 h in serum isolated from vaccinated fish 28 dpi. Data points reflect the log_10_ fold change in CFU relative to input for each serum sample (*n =* 5). Bars show geometric means; the open circle reflects the limit of detection. ***, *P* < 0.001 based on one-way ANOVA and Fisher’s least significant difference (LSD) multiple comparison posttest. (C) Serum antibodies (IgM) against *E. piscicida* at 28 dpi were assayed by ELISA. Data reflect the mean absorbance and SEM (*n =* 5 for each condition). **, *P* < 0.01, based on one-way ANOVA and Fisher’s LSD multiple comparison posttest. (D) Bacterial load in kidneys of vaccinated fish after challenge with WT (Cm^r^) *E. piscicida*. The mean and SEM CFU per gram of tissue are shown (*n =* 5 fish per time point). The dotted line indicates the LOD (200 CFU/g). (E) Survival of vaccinated turbot after challenge with the WT. Fish (*n =* 90 per condition) were challenged 30 days after vaccination and monitored for 28 additional days. ***, *P* < 0.001 comparing LAV vaccine strains with the PBS control using Kaplan-Meier survival analysis with a log rank test (Mantel-Cox). (F) Relative protection index (RPS ± SEM) of each vaccine candidate, based on mortality at 28 dpi for *n =* 3 groups of 30 challenged fish.

Three new mutants, each lacking a single gene from cluster 2 (*pabA*, *pabB*, or ETAE_RS10450) were generated and tested for their ability to produce an immune response protective against *E. piscicida*. Parallel assays were performed using existing vaccine strains (WED and the Δ*aroC* and Δ*esrB* mutants as positive controls) and with formalin-killed WT bacteria (FKC) and phosphate-buffered saline (PBS) treatment (negative controls). The new candidate LAV strains and the previously tested LAV strains did not induce significant mortality compared with controls following i.p. injection of naive fish (see Fig. S4A in the supplemental material). In general, all 6 of the vaccine strains colonized the turbot kidney more robustly than the liver and spleen: with one exception (the ETAE_RS10450 mutant), the vaccine strains were still recoverable from the kidneys but not the spleens or livers of animals 45 dpi (Fig. 5A; Fig. S4C). New and previously validated vaccine candidates induced similar levels of IgM and serum bactericidal activity against *E. piscicida* (Fig. 5B and C), which markedly exceeded those of negative controls. Collectively, these analyses are consistent with the hypothesis that the characteristic fitness dynamics of cluster 2 mutants are suitable for development of LAV strains.

10.1128/mBio.01581-17.5 FIG S4  Safety and bacterial loads of LAV strains in turbot. (A) Survival of turbot (3 months old, 25 ± 3 g) after i.p. injection of ~1.2 × 10^4^ CFU/g of LAV strains (*n =* 30 per strain). Error bars represent SEM among three replicates. (B) Inflammation at injection sites was examined 14 days after challenge of vaccinated turbot with WT *E. piscicida* EIB202. Inflammation was scored as 1 (no symptoms), 2 (lump), 3 (red and swollen), or 4 (fester). Each point represents an individual fish (*n =* 10 for each group). *, *P* < 0.05; **, *P* < 0.01, based on the Kruskal-Wallis statistic with Dunn’s posttest for multiple comparisons. (C) Bacterial loads recovered from spleens and livers of fish i.p. inoculated with the LAV candidates at a dose of 3 × 10^5^ CFU/fish. Each time point reflects the mean and SEM from 5 fish. The dotted line indicates the limit of detection (LOD [200 CFU/g]). (D) Bacterial loads of WT *E. piscicida* recovered from spleens and livers of vaccinated fish. Fish were challenged with WT *E. piscicida* 30 days after vaccination. WT bacteria were detected as Cm^r^ CFU. The mean and SEM CFU per gram of tissue is shown (*n =* 5 fish per time point). The dotted line indicates the LOD (200 CFU/g). Download FIG S4, PDF file, 0.1 MB. Copyright © 2017 Yang et al. 2017 Yang et al. This content is distributed under the terms of the Creative Commons Attribution 4.0 International license.

LAV-immunized and control-treated fish were injected with WT bacteria 30 days after immunization and then monitored for inflammation, colonization by WT bacteria, and survival of the fish. All immunized fish showed no or minimal signs of inflammation at the injection sites (score of 1 or 2), while the PBS/FKC control fish had obvious inflammation (score of 3 or 4) (Fig. S4B). Furthermore, the bacterial loads of WT *E. piscicida* in all the immunized fish declined, whereas they increased in the PBS/FKC control fish (Fig. 5D; Fig. S4D). Finally, the survival of the vaccinated fish exceeded that of PBS/FKC control fish. Death among control fish initiated at day 18, and mortality reached 73.3% at 28 dpi, whereas no fish immunized with the new LAV strains died before 21 dpi, and mortality only reached 13.3 to 16.7% by 28 dpi. Consequently, a markedly higher relative protection ratio was observed for the LAV strains (Fig. 5E and F). In all these assays, the efficacy of the new candidate LAV strains was comparable to or greater than that of previously characterized vaccines. Collectively, these experiments strongly suggest that determination of dynamic fitness patterns may have considerable strategic value to guide development of new live attenuated vaccines.

## DISCUSSION

PACE enables extraction of information from TIS beyond that obtained from traditional endpoint analyses. Here, we demonstrate that application of pattern recognition analysis to time series TIS data increases the sensitivity of comparative fitness assays, facilitating identification of loci with subtle as well as dramatic growth deficiencies. Pattern recognition can also highlight changes in growth conditions or growth requirements that occur during the course of an experiment, through identification of loci whose disruption has temporally determined effects upon growth (i.e., genes fit to second-order equations or higher). Biologically meaningful observations can also be gleaned through clustering of loci according to their growth dynamics. For example, we observed that phenotypically related loci (e.g., components of T3SS or T6SS) clustered together with respect to their growth kinetics and that selection of mutants with a particular pattern of growth attenuation yielded promising new LAV candidates.

A key benefit of PACE analysis versus simple endpoint analysis is that it avoids reliance on arbitrary effect size thresholds. The utility of this approach is evident from inspection of data for T3SS and T6SS. Most genes for both systems meet effect size cutoffs in a statistically significant fashion by 14 dpi, but at earlier points during infection (e.g., 5 dpi), their roles are not always evident based on single endpoint analysis (Fig. 2A). However, PACE analysis of time series data assigns a negative linear coefficient to these genes, suggesting that T3SS and T6SS genes play an important role throughout the majority of infection. PACE also enables identification of genes with consistent but subtle growth deficiencies: e.g., cluster 1 genes (Fig. 4). Since long-term analyses of *in vitro* growth were not performed, it is possible that the effects of the cluster 1 genes are not limited to the *in vivo* environment. Nonetheless, typical endpoint analyses would not have enabled recognition of their contribution *in vivo*.

Although we have applied PACE to analysis of *in vivo* data, it is equally applicable to time series data from other growth environments, such as samples collected at various stages during the growth of *in vitro* batch cultures. Such analyses would allow discrimination between mutants that become underrepresented for distinct reasons, such as growth-phase-dependent fitness alterations or application of selective pressure (e.g., drug treatment). Additionally, performing clustering on combined PACE data from different conditions representing different selective pressures—for instance, drug treatments or different organs—may enable further characterization and refinement of fitness patterns. Moreover, integrating time-resolved, quantitative fitness parameters should have applications outside transposon-based studies; PACE’s analytic framework is equally applicable to forward genetic studies using any method of signature-tagged mutagenesis, including screens based on clustered regularly interspaced short palindromic repeats with Cas9 (CRISPR/Cas9) (37).

TIS analyses that calculate fitness costs per generation (4, 5) presume that growth can be modeled as a linear process across an experiment, and for many mutants, the calculated fitness has been confirmed to be very near their actual growth rate (4, 5, 29, 30). Our analyses support the idea that growth of most mutants can be effectively modeled with either no change or a fairly consistent difference in growth rate (zero-order and first-order equations, respectively) from that of the population average. However, PACE also allowed us to identify a biologically significant subset of genes whose growth was best described using more complex equations, indicative of fitness that varied across the duration of the experiment. For example, fitness profiles of genes modeled by higher-order functions tended to show decreased slopes late in infection, perhaps reflecting infection-related changes in the host, such as (for example) the onset of an adaptive host response. Ultimately, detection of such variance, as well as clustering of genes based upon their fitness profiles, may facilitate insight into the biological roles filled by their products.

## MATERIALS AND METHODS

### Strains, media, and culture conditions.

The bacterial strains, plasmids, and primers used in this study are listed in Tables S3B and S3C. The WT *E. piscicida* strain (formerly known as *E. tarda*) used in this study is EIB202, which was isolated from an outbreak in farmed turbot (8). Culture, cloning, and conjugation were performed using standard conditions; details are presented in Text S1 in the supplemental material. In accordance with biosafety requirements, LAV candidate strains were constructed in the wt_ΔP strain: i.e., EIB202 lacking pEIB202, which carries antibiotic resistance genes but does not contribute to colonization (24).

10.1128/mBio.01581-17.1 TEXT S1  Supplemental methods. Download TEXT S1, DOCX file, 0.1 MB. Copyright © 2017 Yang et al. 2017 Yang et al. This content is distributed under the terms of the Creative Commons Attribution 4.0 International license.

### Transposon mutant library preparation.

The transposon insertion mutant library was generated by conjugation between *E. piscicida* EIB202 (recipient) and SM10 λ*pir*/p*Mar2xT7* (transposon donor); a detailed protocol is presented in Text S1. A fraction of the library was processed for sequencing, and the remaining bacteria were frozen with 20% glycerol for future *in vivo* studies.

### Turbot colonization and survival assays.

All turbot experiments were conducted at the aquaculture station in Yantai, Shandong Province, China, according to protocols approved by Animal Care Committee, East China University of Science and Technology (2006272). The Experimental Animal Care and Use Guidelines from the Ministry of Science and Technology of China (MOST-2011-02) were strictly adhered to. Unless otherwise indicated, experiments were performed with 6-month-old turbot weighing 150 ± 15 g. For pilot experiments, fish (*n =* 30 per dose) were injected intraperitoneally (i.p.) with 3 × 10^5^, 3 × 10^6^, or 3 × 10^7^ CFU/fish, and survival was monitored over the following 30 days. For an additional set of fish (*n =* 5 per time point), fish were anesthetized (10 min) in seawater supplemented with MS-222 (0.02% vol/vol) and then aseptically dissected to harvest liver, spleen, and kidney. Organs were harvested up to 14 dpi, and CFU per gram of tissue were enumerated by plating homogenized tissue on LB agar containing antibiotics. The safety of LAV strains was tested in 3-month-old turbot (25 ± 3 g, ~1.2 × 10^4^ CFU/g i.p. [*n =* 30 per strain]), which were observed for 60 dpi.

For infections using the transposon insertion library, 6-month-old fish were i.p. injected with 100 µl of bacteria (~3 × 10^6^ CFU/fish, or ~2 × 10^4^ CFU/g, with the precise inoculum size determined by plating). At various times after library inoculation, turbots were aseptically dissected to obtain liver, spleen, and kidney. Five organs were combined for each of 3 replicates at each time point. A portion of the bacterial suspension (~1 × 10^5^ to 5 × 10^5^ CFU) from organ homogenates was outgrown overnight on selective LB plates and then recovered and frozen for TIS output library construction.

### Library preparation for TIS.

Libraries for high-throughput sequencing were constructed as previously described (18) with slight modifications. Details are presented within Text S1.

### High-throughput sequencing, identification of essential loci, and functional gene classification.

High-throughput sequencing was performed on Illumina MiSeq or HiSeq 2500 platforms (Illumina, San Diego, CA) and yielded 2 to 3 million reads for each library. Details on the sequencing protocol, read processing, and mapping to the EIB202 chromosome (CP001135) are presented in Text S1. Reads per TA site were tallied and assigned to annotated genes or intergenic regions as in reference 20. Essential loci were determined using the HMM module of EL-ARTIST (window size of 10 TA sites; *P* value of 0.01) (18). Since the sliding window size was set at 10 TA sites, we have reduced confidence in calls made for genes that are smaller than the window size itself, as these are more likely to evade insertion by chance. Functional classification is based on the 2014-updated COG database (31), following the COG software’s protocol (32). Statistical analysis of COG representation was performed using bootstrapping and a 95% confidence interval corrected for multiple testing using the Benjamini-Hochberg procedure (33). KEGG pathway analysis was performed with Kobas 2.0 (34).

### Endpoint-based identification of CE loci.

As previously described (20), reads for each output library were normalized based on the input library. The average of triplicates and the variance were computed for each time point. The fold change (FC) and Mann-Whitney *U* test (MWU) of each locus are based on comparison of the output and input libraries. Endpoint conditionally essential (CE) genes were defined as having a log_2_ FC lower than −2 and an MWU *P* value of <0.05 in at least two of three replicates.

### Time series analysis and gene clustering (PACE).

To apply PACE and identify *in vivo* decreasing (IVD) genes based on the time series data set, we first discarded genes classified by EL-ARTIST as essential, genes with fewer than 50 reads in the input library, and genes for which we identified reads in less than 25% of samples, as the latter data are too sparse for curve fitting. After normalization and filtering, reads were log_2_ transformed and the time course fit to a series of polynomial models (up to and including cubic order) using weighted least-squares regression. Weights were set equal to the inverse variance measured at each time point among the replicates; for time points in which no variance was available, the weight was set equal to the minimum measured weight. Models were selected using the *F* test from the R analysis of variance (ANOVA) functionality, and a cutoff of *P* = 0.05 was used to decide whether to proceed to a more complex model. Clustering analysis was performed in MatLab R2016b (the MathWorks, Inc., Natick, MA). Fit parameters for each IVD locus were clustered using the MatLab’s hierarchical clustering functions, using the Mahalanobis distance metric and the “average” linkage option. Related MatLab and R scripts are available at https://bitbucket.org/gabriel_billings/pace.

### Validation of TIS analysis with *E. piscicida* deletion mutants.

Deletion mutants, along with the WT and wt_ΔP strains, were barcoded with unique 16-bp random sequence tags (35) (Table S3A), which were inserted downstream of the neutral site *glmS* (ETAE_RS16565) (36). Tagged strains (two per mutant) were pooled at equal abundances, and infection, harvesting of organs, and plating were carried out as described for the transposon studies. Barcode regions within genomic DNA (gDNA) recovered from colonies were amplified by PCR, tagged for multiplexing, and sequenced on the Illumina MiSeq platform. Each library yielded 50,000 to 100,000 reads. The competitive index for each mutant at each time point was calculated (18) by comparing the relative numbers of mutant barcode and WT sequences in the recovered samples and the inoculum (*n =* 3 per time point).

### Immunization and challenge.

Bacterial suspensions of vaccine strains prepared as described above for injection were i.p. injected (~3 × 10^5^ CFU/fish) into 3-month-old turbot (25 ± 3 g; ~1.2 × 10^4^ CFU/g). Formalin-killed bacteria and PBS were also injected as negative controls. Organs were harvested from a subset of fish (*n =* 5 per time point) for assessment of CFU, as described above. For challenge experiments, 4-month-old fish were inoculated intramuscularly (i.m.), as in previous studies (21), with 2 × 10^3^ CFU of the WT/fish (approximately 2× the 50% lethal dose [LD_50_] for i.m. injection). All challenge tests were performed in triplicate with 30 fish for each group. The mortality of challenged fish was recorded daily for 28 days after inoculation, and the relative protection ratio (RPS) of the vaccinated group was calculated as follows: RPS = 100% × [1 − (mortality of vaccinated fish/mortality of control fish)]. Additionally, WT colonization within livers, spleen, and kidney (CFU per gram) was determined at 5, 10, 15, 20, and 28 dpi (*n =* 5 fish per time point).

### Serum bactericidal activity.

At 28 days postvaccination, serum was harvested (Text S1) from 6 fish of each vaccine candidate and the control group. To test bactericidal activity, 270 µl of serum was mixed with 30 µl 2 × 10^6^ CFU/ml WT *E. piscicida* and incubated at 30°C. Bacterial CFU were assessed 8 h after inoculation (*n =* 3).

### ELISA.

Serum antibodies against *E. piscicida* were measured using enzyme-linked immunosorbent assays (ELISAs) (21) and microtiter plates coated with formalin-killed WT cells (FKC). After the blocking, incubation, and washing steps (Text S1), wells were incubated with 3,3′,5,5′-tetramethylbenzidine (TMB) solution (Tiangen, Beijing, China) as a color-developing substrate. Reactions were terminated by addition of 5 µl 2 M H_2_SO_4_, and absorbance at 450 nm was assayed using a microplate reader (Bio-Rad, Hercules, CA).

### Availability of data.

Sequencing data from this study have been submitted to the NCBI Sequence Read Archive (SRA; http://www.ncbi.nlm.nih.gov/sra) under accession no. SRR5690733 to SRR5690805.
