# Supplementary figures and images for: Time-Resolved Transposon Insertion Sequencing Reveals Genome-Wide Fitness Dynamics during Infection
Source: mBio. 2017 Oct 3;8(5):e01581-17. doi: 10.1128/mBio.01581-17 (PMC5626973; doi:10.1128/mBio.01581-17)

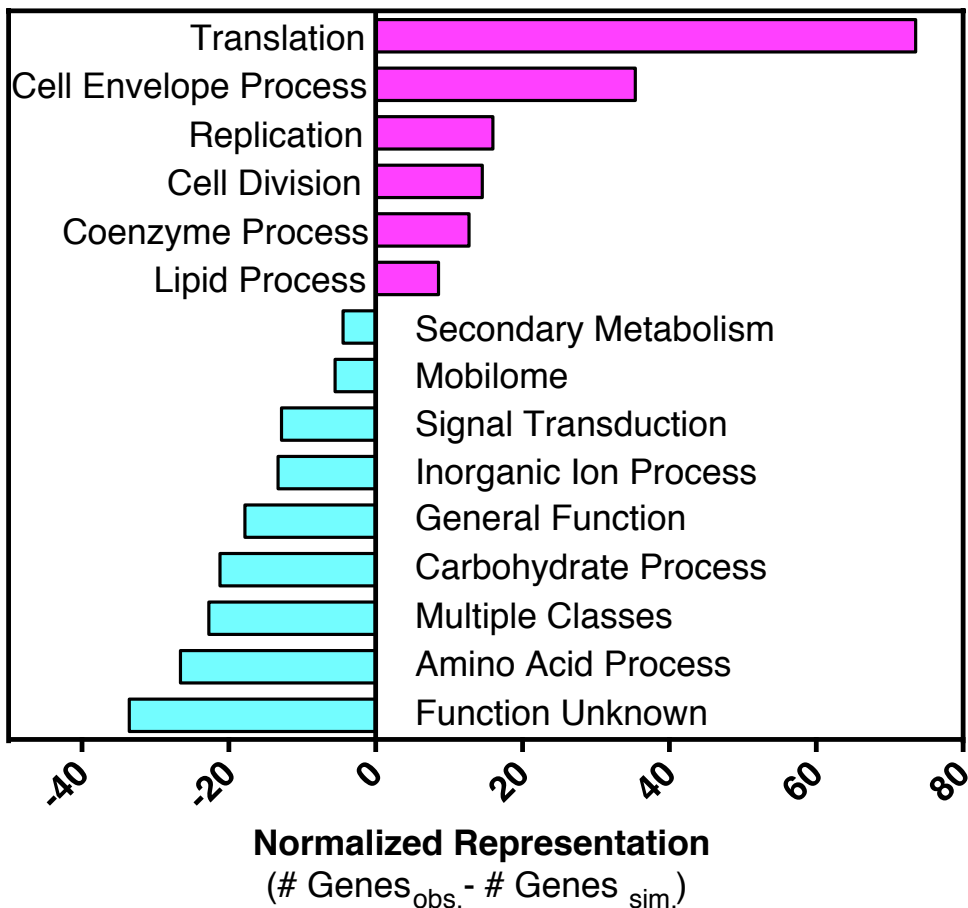

Supplement: FIG S1 [file mbo005173518sf1.pdf]

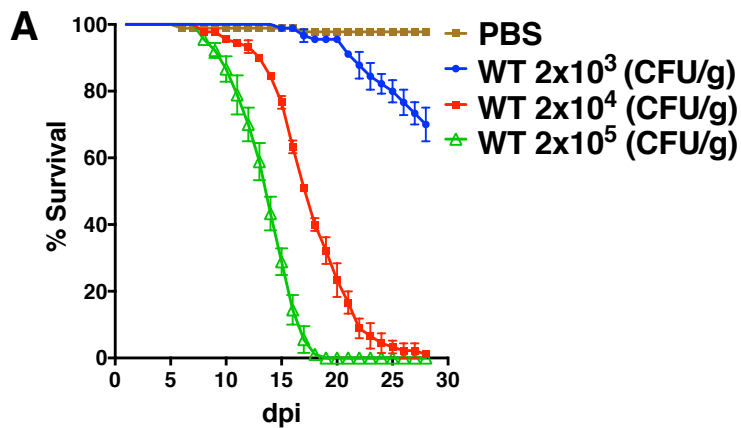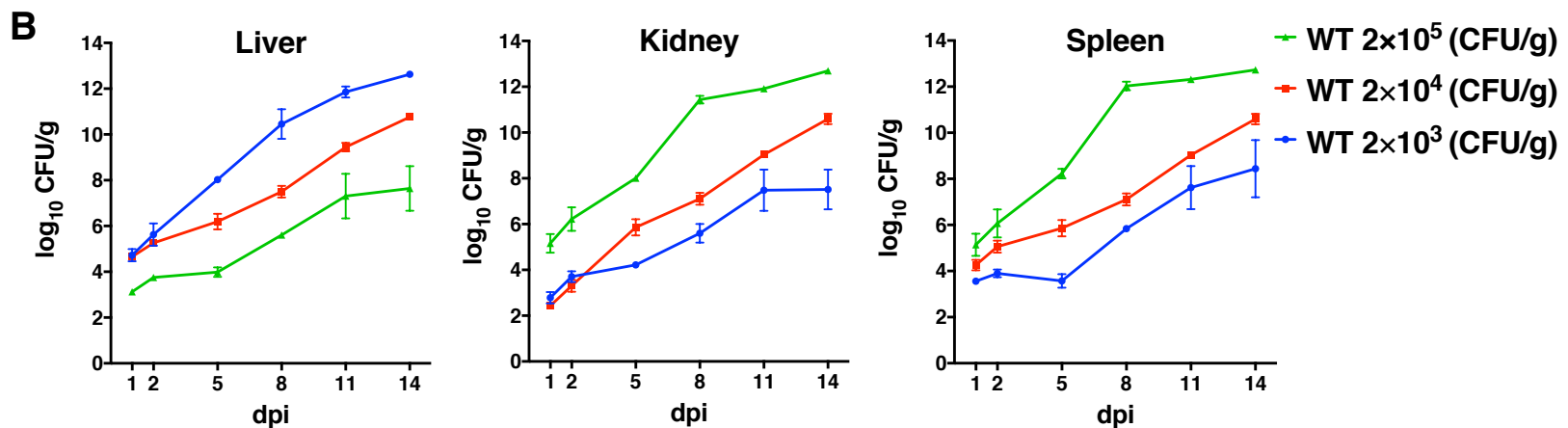

Supplement: FIG S2 [file mbo005173518sf2.pdf]

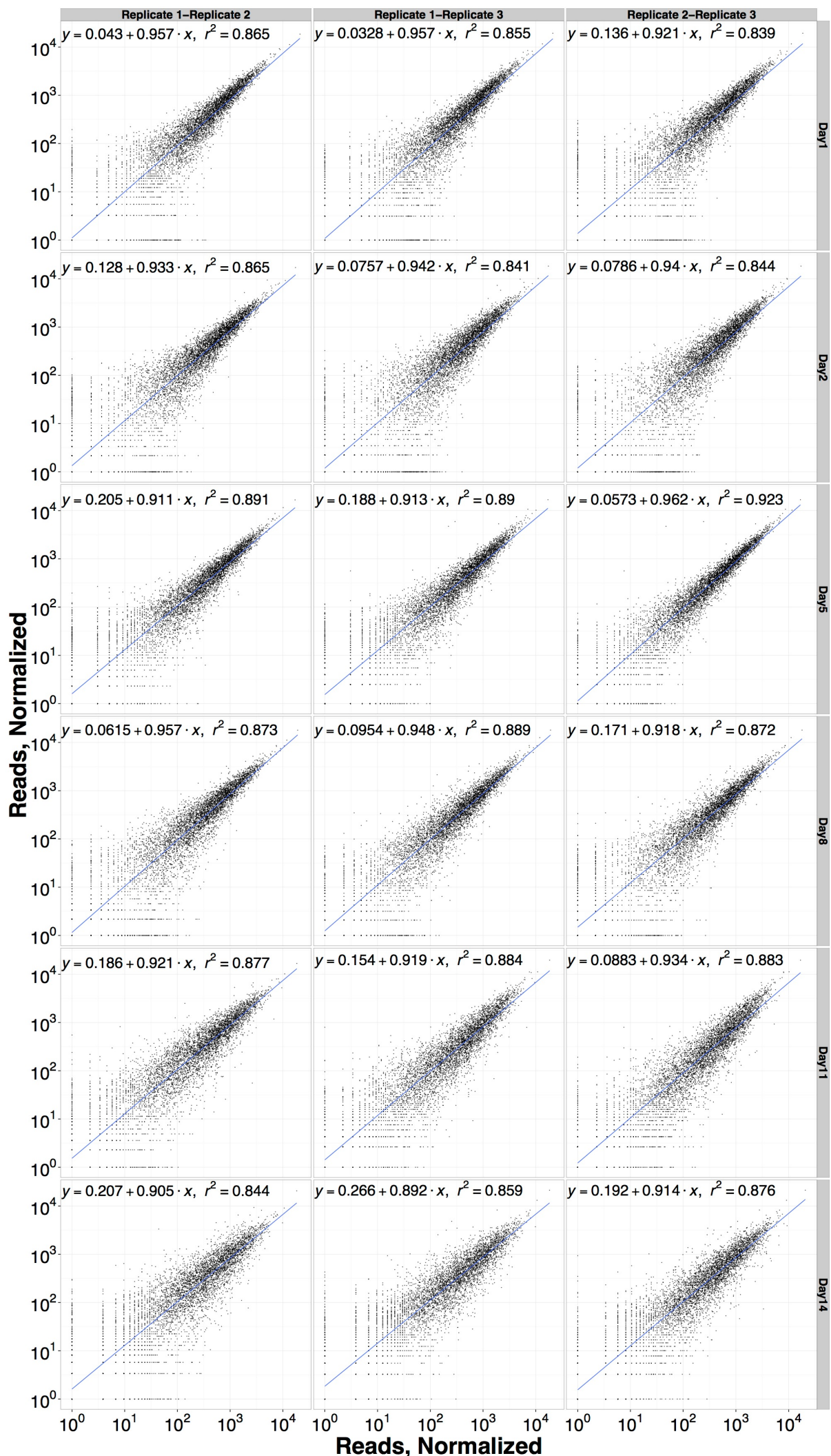

Supplement: FIG S3 [file mbo005173518sf3.pdf]

**A**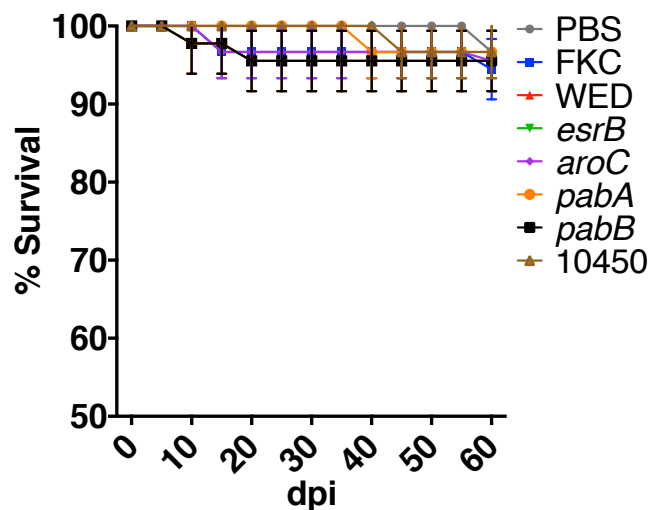**B**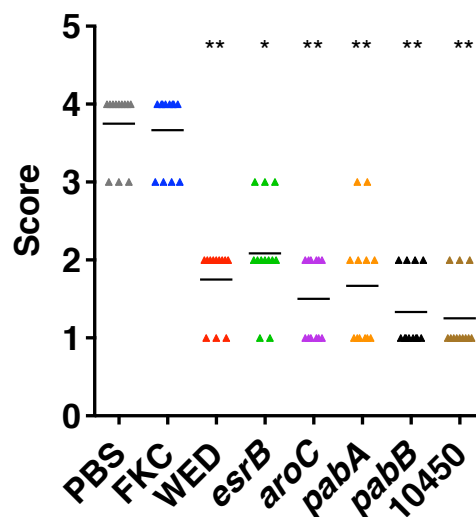**C**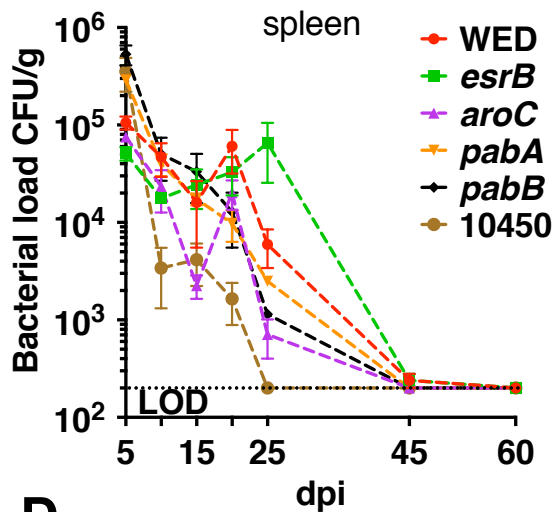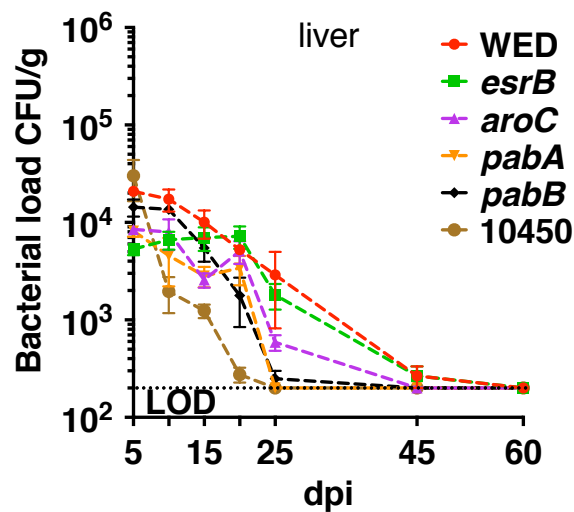**D**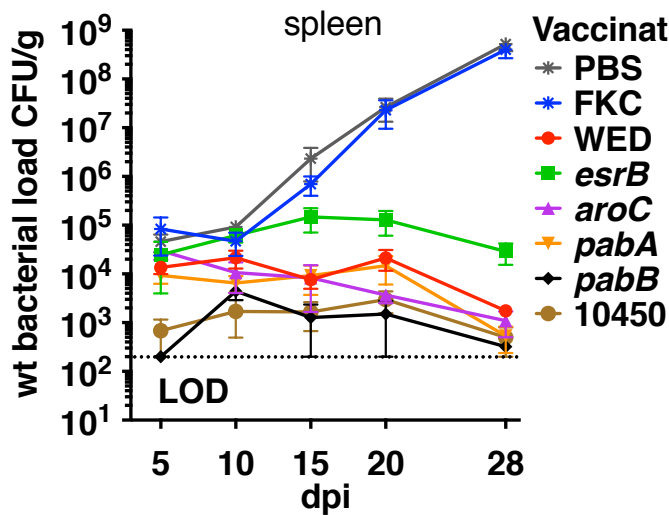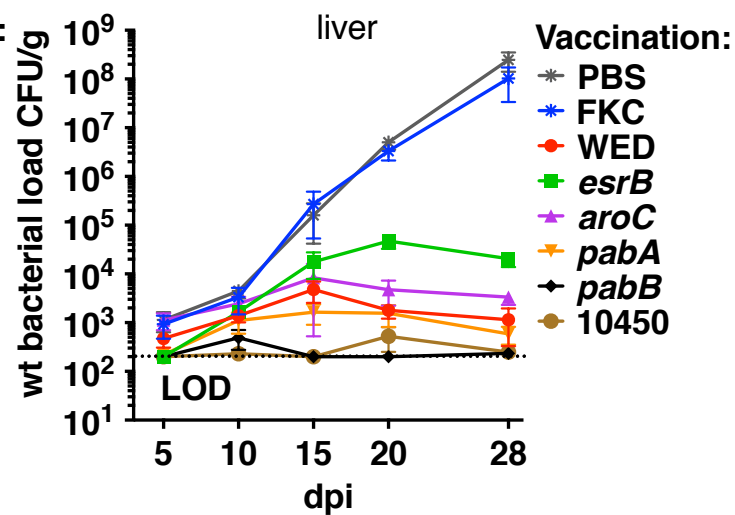

Supplement: FIG S4 [file mbo005173518sf4.pdf]
