# Supplementary material for: Time-Resolved Transposon Insertion Sequencing Reveals Genome-Wide Fitness Dynamics during Infection
Source: mBio. 2017 Oct 3;8(5):e01581-17. doi: 10.1128/mBio.01581-17 (PMC5626973; doi:10.1128/mBio.01581-17)
Supplement: TEXT S1 [file mbo005173518s1.docx]

**Time resolved transposon insertion sequencing reveals genome-wide fitness dynamics during infection**

## SUPPLEMENTAL METHODS

### Strains, media, and culture conditions

Bacterial strains, plasmids, and primers used in this study are listed in Supplemental Tables S3B and S3C. The wt *E. piscicida* strain (formerly known as *E. tarda*) used in this study is EIB202, which was isolated from an outbreak in farmed turbot (1). *Escherichia coli* CC118 *λpir* was used for molecular cloning and *E. coli* SM10 *λpir* was used for conjugation. *E. piscicida* was cultured at 30°C in Luria-Bertani (LB) supplemented with colistin. *E. coli* was cultured at 37°C in LB. Antibiotics were used at the following concentrations: colistin (Col, 12.5 µg/ml), gentamycin (Gm, 75 µg/ml) and chloramphenicol (Cm, 25 µg/ml). *E. piscicida* in-frame deletion mutants were constructed using derivatives of the suicide plasmid pDM4 (2) and sucrose-based counter-selection as described previously (3). The junctions of each in-frame deletion were confirmed using DNA sequencing. In accordance with biosafety requirements, LAV candidate strains were constructed in wt_ΔP, i.e. EIB202 lacking pEIB202, which carries antibiotic resistance genes but does not contribute to colonization (4).

### Transposon mutant library preparation

The transposon insertion mutant library was generated by conjugation. Mutants from 30 conjugation reactions were pooled to generate the transposon library. For each conjugation reaction, pelleted and rinsed cells from 500 µl overnight stationary phase *E. piscicida* EIB202 (recipient) and 300 µl overnight stationary phase SM10 *λpir*/p*Mar2xT7* (transposon donor) were resuspended in 100 µl LB broth. Suspensions were spotted onto 0.45 µM filters (Sartorius, German) on LB agar plates, then incubated at 30°C for 3 hours. Bacteria were resuspended in LB and plated onto LB agar supplemented with Col and Gm. After 24 h incubation at 30°C, scraped colonies (approximately 10,000 CFU per reaction) were resuspended in LB, and then passaged overnight twice, with a 10,000 fold expansion (~13 generations). The outgrown library was processed for sequencing that enabled essential gene analysis, or applied for turbot infection experiments. The remaining bacteria were frozen with 20% glycerol for future studies.

### Turbot colonization and survival assays

All turbot experiments were conducted at the aquaculture station in Yantai, Shandong province of China, according to protocols approved by Animal Care Committee, East China University of Science and Technology (2006272). The Experimental Animal Care and Use Guidelines from Ministry of Science and Technology of China (MOST-2011-02) was strictly adhered to. Unless otherwise indicated, experiments were performed with 6-month old turbot weighing 150±15 g, which were maintained in aerated tanks supplied with a continuous flow of clear deep seawater at 15±1°C. Fish were fasted 48 h before and after the inoculation. For pilot experiments, fish (*n*= 30 per dose) were injected intraperitoneally (IP) with 3×10^5^, 3×10^6^ or 3×10^7^ CFU/fish resuspended in phosphate buffered saline (PBS) and survival was monitored over the following 30 days. For an additional set of fish (*n* = 5 per time point), fish were anesthetized (10 min) in sea water supplemented with MS-222 (0.02% v/v), then aseptically dissected to harvest liver, spleen, and kidney. Organs were harvested up to 14 days post infection (dpi), and CFU/g tissue was enumerated by plating homogenized tissue on LB agar containing antibiotics. The safety of LAV strains was tested in 3-month-old turbot (25±3 g, ~1.2×10^4^ CFU/g via IP route) (*n*= 30 per strain), which were observed for 60 dpi.

For infections using the transposon insertion library, 200 µl of the library stock was plated on LB agar supplemented with Col and Gm, and incubated overnight at 30°C. Colonies were then scraped off of the plates, washed twice with 0.9% saline (w/v), and diluted to ~3×10^7^ CFU/ml. Six-month-old fish were IP injected with 100 µl of bacteria (~3×10^6^ CFU/fish, or ~2×10^4^ CFU/g). The precise inoculum size was determined by plating, and the remaining bacteria were frozen with 20% glycerol for construction of the TIS input library.

At various times after library inoculation, turbots were aseptically dissected to obtain livers, spleen and kidney. Five livers, spleens, or kidneys were combined for each of 3 replicates at each time point. Homogenized tissue was centrifuged (100 RCF) to remove large particles and eukaryotic cells, then 1/5 to 1/2 of the homogenate (~ 1×10^5^ to 5×10^5^ CFU) was plated on selective LB plates and incubated at 30^o^C overnight. Colonies were then washed with PBS/20% glycerol and frozen for TIS output library construction.

### Library preparation for TIS

Libraries for high-throughput sequencing were constructed as previously described (5) with slight modifications. Briefly, for each library, genomic DNA (gDNA) from 5 OD of bacteria was extracted using gDNA extraction columns (Tiangen, Beijing, China). gDNA (5 µg) was diluted in 100 µl TE, and sonicated in a Bioruptor (Diagenode, Belgium) with 30s ON/ 60s OFF for 12 cycles, yielding 200 ~ 800 bp fragments. End repair and 3’ end A-tailing was performed with 1 µg sheared DNA and the VAHTS turbo DNA library preparation kit (Vazyme, Nanjing, China), and adaptor ligation was performed with forked adaptors (6). Two rounds of PCR amplification were performed to enrich for sequences adjacent to transposon insertion sites and attach Illumina P5 and P7 hybridization sequences and barcodes for multiplexing. Final products (300 to 500 bp) were isolated from a 2% agarose gel and extracted with a gel purification column (Qiagen, Germany).

### High-throughput sequencing, identification of essential loci and functional gene classification

High-throughput sequencing was performed on Illumina MiSeq or HiSeq 2500 platforms (Illumina, San Diego, CA, USA). The sequencing protocol was 65-cycle single end read plus 7-cycle index read. Sequencing generated 2 to 3 million reads for each library. The raw data were processed to remove adaptor sequences by CutAdapt software (7), and then mapped to the *E. piscicida* EIB202 chromosome (CP001135) using Bowtie (8). After mapping, the reads per TA site were tallied and assigned to annotated genes or intergenic regions using EL-ARTIST (5). Essential loci were determined using the HMM module of EL-ARTIST (window size of 10 TA sites, *P*-value of 0.01) (5). Functional classification is based on the 2014-updated COG database (9), following the COG software’s protocol (10). Statistical analysis of COG representation was performed using bootstrapping and a 95% confidence interval corrected for multiple testing using the Benjamini-Hochberg procedure (11). KEGG pathway analysis was performed with Kobas 2.0 (12).

### End point-based identification of conditionally essential loci

As previously described (6), reads for each output library were normalized based on the input library. The average of triplicates at each time point was computed, as was the variance at each time point. The fold change (FC) and Mann Whitney *U* test of each loci were calculated by comparing the output and input libraries. The end-point conditional essential (CE) genes were defined as log_2_FC lower than -2, and MWU *P* value lower than 0.05 in at least two replicates among the triplicated experiments.

### Time series analysis and gene clustering (PACE)

To apply PACE and classify *in vivo* decreasing (IVD) category of the genes based on time-series dataset, we first discarded genes classified by EL-ARTIST as essential and genes for which we identified reads in less than 25% of samples. After normalization and filtering, reads were log_2_ transformed and the time course fit to a series of polynomial models (up to and including cubic order) using weighted least-squares regression. Weights were set equal to the inverse variance measured at each time point among the replicates; for time points in which no variance was available, the weight was set equal to the minimum measured weight. Models were selected using the F-test from the R ANOVA functionality, and a *P* = 0.05 cutoff was used to decide whether to proceed to a more complex model. Clustering analysis was performed in MATLAB R2016b (The Mathworks, Inc., Natick, Massacusetts, USA). Fit parameters for each IVD locus were clustered using the MATLAB’s hierarchical clustering functions, using the Mahalanobis distance metric and the ‘average’ linkage option. Related MATLAB and R scripts are available at https://bitbucket.org/gabriel_billings/pace.

### Validation of TIS analysis with *E. piscicida* deletion mutants

Deletion mutants, along with wt and wt_ΔP*,* were barcoded with unique 16-bp random sequence tags (13) (Supplemental Table S3A) inserted downstream of *glmS* (ETAE_RS16565), a site that could be disrupted without *in vitro* or *in vivo* fitness effects (14). For each mutant, duplicate strains were independently tagged with two distinct barcodes. Tagged strains were pooled at equal abundances, and infection, harvesting of organs, and plating was carried out as described for the transposon studies. The gDNA recovered from colonies was subjected to PCR with primers to amplify the barcode region and introduce Illumina P5 and P7 hybridization sequences and additional barcodes for multiplexing. The libraries were mixed and sequenced on Illumina MiSeq platform. Each library yielded 50,000 to 100,000 reads. The competitive index for each mutant at each time point was calculated (5) by comparing the number of mutant barcode and wt sequences in the recovered samples and the inoculum (*n*=3 per time point).

### Immunization and challenge

Bacterial suspensions of vaccine strains prepared as above for injection were IP injected (~3×10^5^ CFU/fish) into 3-month-old turbot (25±3 g; ~1.2×10^4^ CFU/g). Formalin killed bacteria and PBS were also injected as negative controls. Fish were maintained in 1,000 L tanks at 15±1°C supplied with deep-sea water, and organs were harvested from a subset of fish (*n*=5 per time point) for assessment of CFU, as described above. For challenge experiments, 4-month-old fish were inoculated intramuscularly (IM), as in previous studies (15), with 2×10^3^ CFU/fish (approx. 2×LD_50_ for IM injection) of wt. All challenge tests were performed in triplicate with 30 fish for each group. The mortality of challenged fish was recorded daily for 28 days after inoculation, and the relative protection ratio (RPS) of vaccinated group was calculated as follows:

$$RPS=100\%\times\left( 1-\frac{mortality of vaccinated fish}{mortality of control fish} \right)$$

Additionally, wt colonization within livers, spleen and kidney (CFU/g) was determined at 5, 10, 15, 20, and 28 dpi (*n*=5 fish per timepoint).

### Serum bactericidal ability

At 28-day post vaccination, 6 fish of each vaccine candidate and control group were picked for testing. The peripheral blood was drawn from the caudal vein of each fish (15). After clotting at 4°C for 1 h, serum was separated by centrifugation at 4,000 *g* at 4°C for 5 min, and then stored at -80°C for serum bactericidal and ELISA tests. To test bacterial survival in serum, 30 µl 2×10^6^ CFU/ml wt suspension was mixed with 270 µl of serum and incubated at 30°C. Bacterial survival (CFU) was assessed 8 h after inoculation (*n*=3).

### Enzyme-linked immunosorbant assay (ELISA)

Serum antibodies against *E. piscicida* were measured using ELISA assays (15). Microtiter plate wells were coated with 100 µl of formalin-killed cells (FKC) of wt overnight at 4°C. Excess cells were discarded, and wells were blocked with 100 µl of PBS containing 2% bovine serum albumin (BSA) for 3 h at 22°C. After removing the blocking solution and washing three times with PBS + 0.05% Tween-20 (PBST), the wells were incubated for 3 h at 22°C with 100 ml of serially diluted turbot serum. The plates were washed and incubated with 100 µl of mouse anti-turbot IgM monoclonal antibody (Aquatic Diagnostic, Stirling, UK) for 1 h, washed 5 times, and further incubated with 100 µl of goat anti-mouse IgG-HRP (Tiangen, Beijing, China) (1:1000) for 1 h at 22°C. Finally, the wells were washed three times followed by addition of TMB solution (Tiangen, Beijing, China) as a color-developing substrate. Reactions were terminated by addition of 5 µl 2 M H_2_SO_4_, and absorbance at 450 nm was assayed using a microplate reader (Bio-Rad, Hercules, CA).

**References**

1. Wang, Q.Y., Yang, M.J., Xiao, J.F., Wu, H.Z., Wang, X., Lv, Y.Z., Xu, L.L., Zheng, H.J., Wang, S.Y., Zhao, G.P., Liu, Q. & Zhang, Y.X. Genome sequence of the versatile fish pathogen *Edwardsiella tarda* provides insights into its adaptation to broad host ranges and intracellular niches. *PLoS ONE* **4**, e7646–16 (2009).
2. Wang, S.Y., Lauritz, J., Jass, J. & Milton, D.L. A ToxR homolog from *Vibrio anguillarum* serotype O1 regulates its own production, bile resistance, and biofilmformation. *J. Bacteriol.* **184**, 1630–1639 (2002).
3. Lv, Y.Z., Xiao, J.F., Liu, Q., Wu, H.Z., Zhang, Y.X. & Wang, Q.Y. Systematic mutation analysis of two-component signal transduction systems reveals EsrA-EsrB and PhoP-PhoQ as the major virulence regulators in *Edwardsiella tarda*. *Vet. Microbiol.* **157**, 190-199 (2012).
4. Zheng, J.Y., Xu, L.L., Wang, Q.Y. & Xiao, J.F. Elimination of multidrug resistant plasmid pEIB202 in fish pathogenic *Edwardsiella tarda*. *J. Anhui Agri. Sci.* **39**, 17944–17948 (2011).
5. Pritchard, J.R., Chao, M.C., Abel, S., Davis, B.M., Baranowski, C., Zhang, Y.J., Rubin, E.J. & Waldor, M.K. ARTIST: High-Resolution Genome-Wide Assessment of Fitness Using Transposon-Insertion Sequencing. *PLoS Genet.* **10**, e1004782 (2014).
6. Chao, M.C., Pritchard, J.R., Zhang, Y.J., Rubin, E.J., Livny, J., Davis, B.M. & Waldor, M.K. High-resolution definition of the *Vibrio cholerae* essential gene set with hidden Markov model-based analyses of transposon-insertion sequencing data. *Nucleic Acids Res.* **41**, 9033–9048 (2013).
7. Martin, M. Cutadapt removes adapter sequences from high-throughput sequencing reads. *EMBnet J.* **17**, 10–12 (2012).
8. Langmead, B., Trapnell, C., Pop, M. & Salzberg, S.L. Ultrafast and memory-efficient alignment of short DNA sequences to the human genome. *Genome Biol.* **10**, R25. (2009).
9. Galperin, M.Y., Makarova, K.S., Wolf, Y.I. & Koonin, E.V. Expanded microbial genome coverage and improved protein family annotation in the COG database. *Nucleic Acids Res.* **43**, D261–269 (2015).
10. Kristensen, D.M., Kannan, L., Coleman, M.K., Wolf, Y.I., Sorokin, A., Koonin, E.V. & Mushegian, A. A low-polynomial algorithm for assembling clusters of orthologous groups from intergenomic symmetric best matches. *Bioinformatics* **26**, 1481–1487 (2010).
11. Benjamini, Y. & Hochberg, Y. Controlling the false discovery rate: a practical and powerful approach to multiple testing. *J. R. Statist. Soc. B* **57**, 289-300 (1995).
12. Xie, C. Mao, X. Huang, J. Ding, Y. Wu, J. Dong, S. Kong, L. Gao, G. Li, CY. Wei, L. KOBAS 2.0: a web server for annotation and identification of enriched pathways and diseases. *Nucleic Acids Res.* **39**, W316–W322 (2011).
13. Abel, S., Abel zur Wiesch, P., Chang, H.H., Davis, B.M., Lipsitch, M. & Waldor, M.K. Sequence tag-based analysis of microbial population dynamics. *Nat. Methods* **12**, 223-226 (2015).
14. Koch, B., Jensen, L.E. & Nybroe, O. A panel of Tn7-based vectors for insertion of the *gfp* marker gene or for delivery of cloned DNA into Gram-negative bacteria at a neutral chromosomal site. *J. Microbiol. Methods* **45**, 187-95 (2001).
15. Xiao, J.F., Chen, T., Liu, B., Yang, W.M., Wang, Q.Y., Qu, J.B. & Zhang, Y.X. *Edwardsiella tarda* mutant disrupted in type III secretion system and chorismic acid synthesis and cured of a plasmid as a live attenuated vaccine in turbot. *Fish Shellfish Immuol*. **35,** 632-641 (2013).
